# Prognostic factors in children with head and neck rhabdomyosarcoma: A 12‐year retrospective study

**DOI:** 10.1002/brb3.1697

**Published:** 2020-06-16

**Authors:** Yi Zhang, Wei‐Ling Zhang, Dong‐Sheng Huang, Yi‐Zhuo Wang, Hui‐Min Hu, Yan‐Yan Mei, Tian Zhi

**Affiliations:** ^1^ Department of Pediatrics Beijing Tongren Hospital Capital Medical University Beijing China

**Keywords:** children, head and neck, prognostic factors, rhabdomyosarcoma, survival

## Abstract

**Introduction:**

To identify possible prognostic factors in children with head and neck rhabdomyosarcoma (RMS).

**Methods:**

A total of 98 patients with head and neck RMS were enrolled in this retrospective study from February 2005 to September 2017. Prognostic factors were evaluated by univariate and multivariate analysis using Cox's proportional hazards model. Survival curves were calculated by Kaplan–Meier method.

**Results:**

At the study closing date, there were 60 patients alive, 37 patients died, one patient was lost to follow‐up, and 47 patients relapsed. The median disease‐specific survival was 60.00 ± 25.36 months, and the overall survival (OS) rate was 61.9%. Complete remission was associated with a longer disease‐specific survival (86.6%) compared with partial remission (6.7%). In addition, patients with age >3 years had better OS rate (69.0%) compared with age ≤3 years (42.3%). Univariate and multivariate analysis showed that chemotherapy efficacy and age were prognostic factors of disease‐specific survival.

**Conclusions:**

Improvement in outcome was obtained with comprehensive treatment for head and neck RMS. Both chemotherapy efficacy and age of patients were prognostic factors for children with head and neck RMS, which provide some valuable information for further treatment.

## Graphical abstract

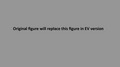

## 1 INTRODUCTION

Rhabdomyosarcoma (RMS) is the most common soft tissue sarcomas of childhood, accounting for approximately 3% of childhood malignancies and with the overwhelming majority of patients presenting by 14 years of age (Córdoba & Inarejos, 2016; Dasgupta, Fuchs, & Rodeberg, 2016; Okcu, Hicks, & Horowitz, 2016). RMS originates from the embryonal mesenchyme that eventually gives rise to striated skeletal muscle, which is further classified as embryonal, alveolar, spindle cell/sclerosing, and pleomorphic histologic subtypes since 2013 (Christopher, Fletcher, & Krishnan, 2013; Perez et al., 2011). Approximately 30% of RMS occurred in head and neck region, commonly divided into para‐meningeal, nonorbital and nonmeningeal, and orbit subsites (Simon, Paulino, Smith, & Buatti, 2002). Many patients cannot be discovered early, and the complete surgical resection is quite difficult, so the recurrence and metastasis are commonly seen in patients and the mortality rate is high (Zhang et al., 2013).

The cure rate of children with RMS has improved remarkably from 25% in 1970 to over 70% with contemporary therapy, due to systematic use of multimodal therapy (surgery, radiation therapy, and chemotherapy) (Crist et al., 1990; Joshi et al., 2004). However, the prognosis of advanced cases with head and neck RMS is poor, the 5‐year survival rate is only 30%–50% (Zhang et al., 2013). In addition, due to the relative rarity of this tumor occurred in head and neck region, institutional experience is usually limited, with only a few literature reports (Simon et al., 2002). Besides, there is a certain gap in the treatment effect between abroad and in China, and the 5‐year survival rate in China is only 14.7%–50% (Zhang et al., 2013). Since 2005, our center has carried out comprehensive treatment with head and neck RMS and it has rich experience in biopsy, operation timing, and individualized treatment (Huang & Zhang^2012^; Zhang et al., 2013). In this study, we aimed to identify possible prognostic factors for children with head and neck RMS treated in our hospital and provide some valuable information for further treatment.

## 2 MATERIALS AND METHODS

### 2.1 Patients

We retrospectively reviewed 98 pathologically diagnosed RMS patients whose primary site was in the head and neck region in our hospital from February 2005 to September 2017. The inclusion criteria were as follows: (a) patients diagnosed with head and neck RMS for the first time in our hospital; (b) patients with age <14 years; and (c) patients undergoing surgery, chemotherapy, radiotherapy, and follow‐up according to comprehensive treatment of our hospital. Patients who died within 3 months of diagnosis and did not undergo surgical treatment were excluded from this study. This study was approved by the ethics committee of Beijing Tong Ren Hospital. Written informed consent was obtained from all participants and their guardians.

### 2.2 Diagnostic criteria

Pathological diagnosis was based on surgical resection or tumor biopsy. Histology was mainly classified as embryonal (tumor cells tend to be more elongated and less densely cellular) and alveolar subtypes (tumor cells tend to be smaller and rounder, often with a denser cellularity) (Wexler, Meyer, & Helman, 2011). Risk stratification (Malempati & Hawkins, 2012) (low risk, intermediate risk, and high risk) for RMS was based on both a pretreatment (TNM) staging system (stage 1–4) and a surgical/pathologic clinical grouping system (group I–IV) established by the IRSG (Lawrence, Anderson, Gehan, & Maurer, 1997; Maurer et al., 1988).

### 2.3 Therapy

Patients received comprehensive treatment. For patients whose tumor could be removed completely, one‐stage surgery was performed firstly, and then, 8‐cycle chemotherapy was carried out after the complete surgical resection. For patients whose tumor invaded more sites and could not be removed completely, tumor biopsy was performed, and then, 2‐ to 4‐cycle chemotherapy was carried out after obtaining pathological diagnosis. In the case of tumor reduction, the second surgery was performed to remove tumor to the maximum extent, and then, 6‐cycle chemotherapy was carried out. Radiotherapy was performed for children over 3 years old (including 3 years old) with group III–IV, and the dose of radiotherapy was 36–45 Gy. Patients with recurrence and metastasis were treated with individualized therapy (CEM [cyclophosphamide/etoposide/melphalan] conditioning regimen combined with autologous peripheral blood stem cell transplantation [APBSCT]) (Huang et al., 2004; Tang et al., 2001) for 2‐year total cycles. The successful hematopoietic reconstitution criteria were as follows: nucleated cells ≥0.5 × 10^9^/L, platelet ≥20 × 10^9^/L, and hemoglobin ≥80 g/L.

Patients were treated with the following four chemotherapy regimens alternately: vincristine, actinomycin, and cyclophosphamide (VAC); vincristine, doxorubicin, and cyclophosphamide (VDC); isocyclophosphamide and etoposide (IE); and vincristine, topotecan, and cyclophosphamide (VTC).

### 2.4 Analysis of therapeutic effect and follow‐up

The prognosis of patients was indicated by the overall survival (OS). OS was calculated from the day of first admission to hospital to the time of the last follow‐up or death. The evaluation criteria of chemotherapy efficacy were complete remission (CR), partial remission (PR), and progressive disease (PD). CR was defined as disappearance of all known tumor lesions. PR was defined as a reduction of the product of the largest perpendicular diameters of all measurable lesions by more than 50%. PD was defined as the appearance of any new lesion not previously identified or an estimated increase of 25% or more in existing lesions. The patients were followed up to July 2018, with a median follow‐up of 41.5 months (range from 2 to 149 months).

### 2.5 Statistical analysis

All statistical analyses were performed by using SPSS version 20.0 (SPSS Institute). Quantitative data were expressed as means ± standard deviations (*SD*). Qualitative data were expressed as number and percentage. Prognostic factor was evaluated by univariate and multivariate analysis using Cox's proportional hazards model. Survival curves were calculated by Kaplan–Meier method. Statistical significance was set at *p* < .05.

## 3 RESULTS

### 3.1 Baseline characteristics

A total of 98 patients (53 males, 45 females; median age: 5.8 years; range from 2 months to 13.4 years) with head and neck RMS were enrolled in this retrospective study from February 2005 to September 2017. The baseline characteristics of included patients were showed in Table 1. Among the 98 patients, 26 (26.5%) were diagnosed within 3 years old (12 patients were diagnosed within 1 years old), 72 (73.5%) were diagnosed after 3 years old (14 patients were diagnosed after 10 years old), 62 (62.3%) patients were embryonal subtype, and 36 (36.7%) patients were alveolar subtype. Among the 36 patients with alveolar subtype RMS, 12 were diagnosed within 3 years old, 23 were diagnosed after 3 years old, and one was diagnosed with positive for fusion of PAX‐FOXO1 genes (age: 10 years old). Otherwise, tumor sites of 43 (43.9%) patients were in the orbit region, 46 (46.9%) patients in the para‐meningeal region, and 9 (9.2%) patients in nonorbital and nonmeningeal region. In addition, 71 (72.5%) patients were in the clinical group III and 27 (27.5%) patients in group IV. Among the 27 patients with clinical group IV, one was diagnosed within 1 year old, four were diagnosed at 1–3 years old, 21 were diagnosed at 4–9 years old, and 1 was diagnosed after 10 years old. The majority of cases were in intermediate risk (42.9%) and high risk (29.6%).

**TABLE 1:** Baseline characteristics of included patients

Characteristic	Number (*n*, %)
Age (years)	
≤3	26 (26.5)
≤1	12 (12.2)
1–3	14 (14.3)
>3	72 (73.5)
3–10	58 (59.2)
≥10	14 (14.3)
Gender	
Male	53 (54.1)
Female	45 (45.9)
Tumor site	
Orbit	43 (43.9)
Para‐meningeal	46 (46.9)
Nonorbital and nonmeningeal	9 (9.2)
Pathology type	
Embryonal	62 (62.27)
Alveolar	36 (36.73)
Clinical group	
Group III	71 (72.5)
Group IV	27 (27.5)
Risk stratification	
Low risk	27 (27.6)
Intermediate risk	42 (42.9)
High risk	29 (29.6)
Treatment	
Radical surgery	83 (84.7)
Only biopsy	15 (15.3)
Radiotherapy	57 (58.2)
Chemotherapy	97 (99.0)
CEM conditioning regimen combined with APBSCT	3 (3.1)
Diagnosis time (year)	
≤2010	37 (37.8)
>2010	61 (62.2)

Abbreviations: APBSCT, autologous peripheral blood stem cell transplantation; CEM, cyclophosphamide/etoposide/melphalan.

Among the 98 patients, 83 (84.7%) were administered with radical surgery, 15 (15.3%) with only biopsy, 57 (58.2%) with radiotherapy, and 97 (99.0%) with chemotherapy, 3 (3.1%) with CEM conditioning regimen combined with APBSCT.

### 3.2 Treatment outcome

**FIGURE 1:**
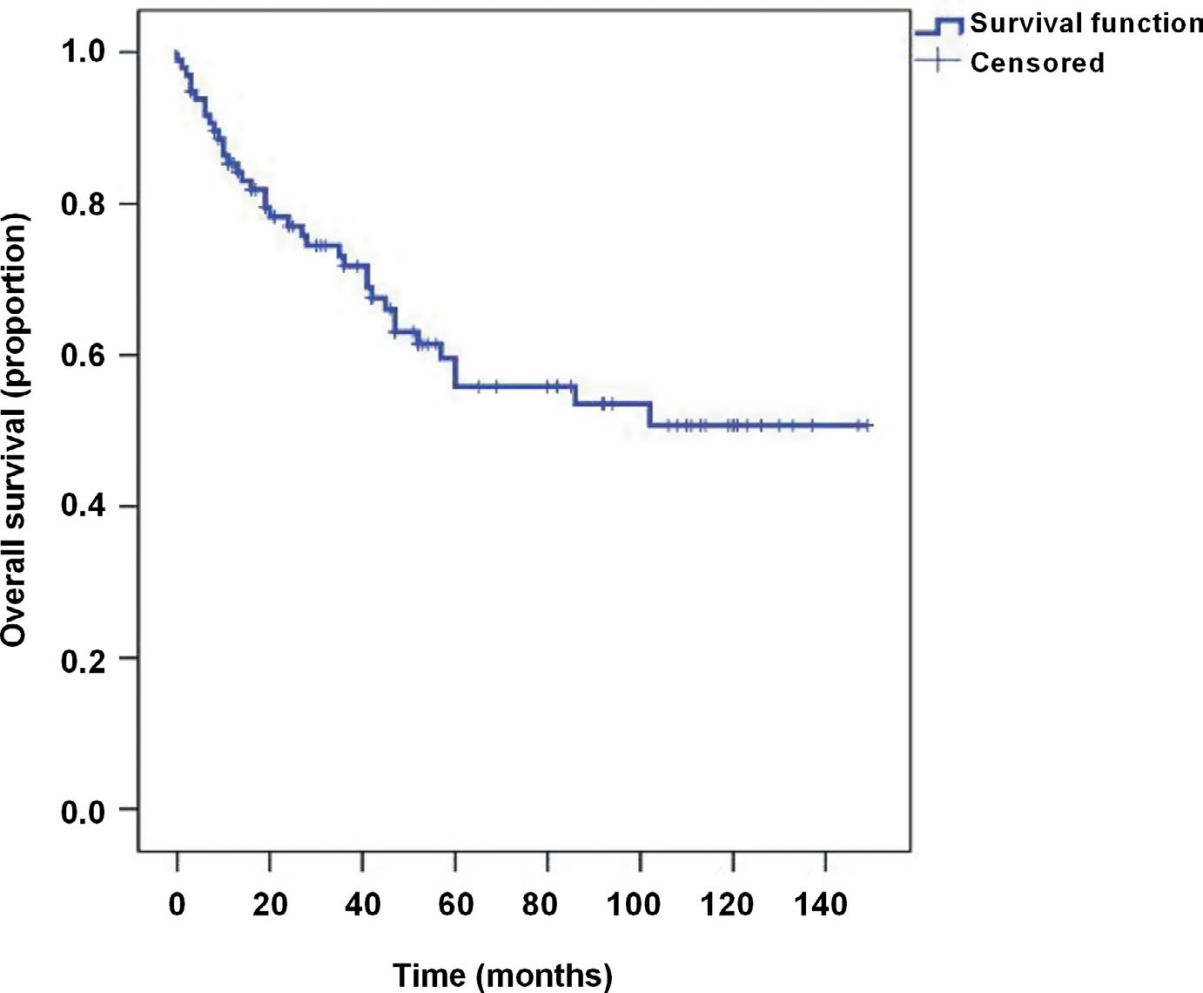
Overall survival of all 98 patients

In our analysis of 98 patients with head and neck RMS, the median follow‐up for survivors was 41.5 months (range, 2–149 months). At the study closing date, there were 60 patients alive, 37 patients had died due to intracranial metastases and tumor progression, one patient was lost to follow‐up, and 47 patients relapsed. In addition, 56 (57.1%) cases obtained the CR, 25 (25.5%) cases obtained the PR, and 17 (17.3%) cases obtained the PD. The median disease‐specific survival was 60.00 ± 25.36 months, and the overall survival rate was 61.9% (Figure 1). In three cases with APBSCT, two cases obtained the CR, one case recurred 6 months after the operation and died 8 months after the recurrence, and the total survival time was 29 months.

### 3.3 Analysis of prognostic factors for disease‐specific survival

**FIGURE 2:**
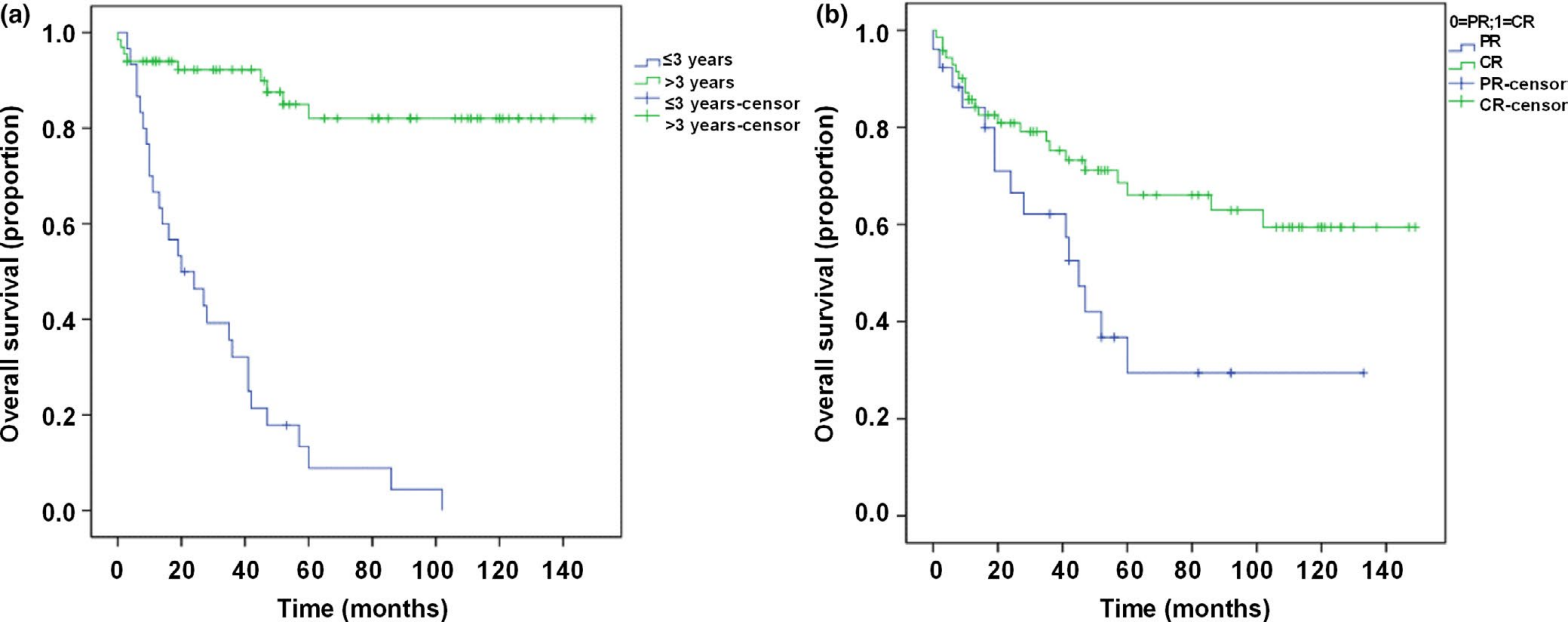
Overall survival according to different categories. (a) Kaplan–Meier survival curves according to age group (age ≤3 and >3 years); (b) Kaplan–Meier survival curves according to chemotherapy efficacy (CR and PR). CR, complete remission; PR, partial remission

The distribution of clinical and pathologic factors that were predictive for disease‐specific survival by both univariate and multivariate analyses was described in Table 2. Univariate analysis revealed that age, pathology type, chemotherapy efficacy, and diagnosis time were significantly associated with survival. The OS rate for patients with age ≤3 and >3 years was 42.3% and 69.0%, respectively (HR = 2.33, *p* < .05) (Figure 2a). CR was associated with a longer disease‐specific survival (OS rate, 86.6%) compared with PR (OS rate, 6.7%) (HR = 16.494, *p* < .05) (Figure 2b). No statistically significant differences in disease‐specific survival were identified when the study group was divided by pathology type (embryonal and alveolar, 61.3% vs. 61.1%) or by diagnosis time (≤2010 year and >2010 year, 62.2% vs. 50.0%). Multivariate analysis showed that age (HR = 0.075; 95% CI: 0.167–4.196; *p* < .05) and chemotherapy efficacy (HR = 0.004; 95% CI: 0.00–0.030; *p* < .05) were predictors of improved disease‐specific survival.

**TABLE 2:** Univariate and multivariate analysis using Cox's proportional hazards model

Independent factors	OS (*n*, %)	Univariate analysis		Multivariate analysis	
		HR (95% CI)	*p* value	HR (95% CI)	*p* value
Age (≤3/>3 years)	11 (42.3)/49 (69.0)	2.33 (1.145–4.754)	.020	0.075 (0.167–4.196)	.003
Chemotherapy efficacy (PR/CR)	2 (6.7)/58 (86.6)	16.494 (6.362–42.764)	.001	0.004 (0.00–0.030)	.001
Diagnosis time (≤2010/>2010 year)	23 (62.2)/37 (50.0)	3.366 (1.312–8.634)	.012	0.922 (0.374–2.275)	.861
Pathology type (embryonal/alveolar)	38 (61.3)/22 (61.1)	0.441 (0.203–0.959)	.039	1.002 (0.414–2.425)	.996
Relapse (no/yes)	18 (38.3)/42 (84.0)	0.463 (0.254–1.865)	.463		
Radiotherapy (yes/no)	35 (62.5)/25 (60.9)	1.251 (0.572– 2.735)	.575		
Clinical group (III/ IV)	46 (65.7)/14 (51.9)	0.239 (0.407–3.869)	.239		
Tumor site					
Nonorbital and nonmeningeal	6 (75.0)	–	.065		
Orbit	32 (74.4)	1.275 (0.248–6.563)			
Para‐meningeal	22 (47.1)	0.373 (0.155–0.897)			
Risk stratification					
High risk	21 (77.8)	–	.413		
Intermediate risk	24 (58.5)	4.374 (0.440–43.528)			
Low risk	15 (51.7)	1.553 (0.517–4.543)			

Abbreviations: 95% CI, 95% confidence interval; CR, complete remission; HR, hazard ratio; OS, overall survival; PR, partial remission.

## 4 DISCUSSION

Rhabdomyosarcoma is a soft tissue sarcoma in pediatric patients, about 30% of which occurs in the head and neck (Chen, Ricciotti, Futran, & Oda, 2017). With the development of comprehensive treatment for RMS, the 5‐year survival rate has been improved to over 70% (Rodeberg & Paidas, 2006). However, there is a certain gap in the treatment effect between abroad and in China, and the 5‐year survival rate in China is only 14.7%–50% (Zhang et al., 2013). In addition, due to the rarity of head and neck RMS, information regarding prognostic factors is limited, with only a few literature reports (Simon et al., 2002). In this study, we aimed to identify possible prognostic factors for patients with head and neck RMS treated in our hospital.

Some researchers had demonstrated that the primary site in the orbit accounts for 25%–35% of patients with RMS in the head and neck region (Badr et al., 2012; Salman et al., 2012; Turner & Richmon, 2011). In this study, the most frequent positions in patients with head and neck RMS were in the orbit and para‐meningeal, which accounted for 43.9% and 46.9%, respectively. It was much higher than that of reported in literature. Some other researchers reported that the peak age incidence of RMS was 7–8 years (Kamimura et al., 2011). In this study, we reported that the median age of incidence was 5.8 years, which consistent with previous reports.

Previous studies had demonstrated that age was an independent prognostic factor in RMS (Joshi et al., 2004; Stiller, Stevens, Magnani, Corazziari, & EUROCARE Working Group, 2001). Joshi et al. (2004) reported that infants less than age 1 and adolescents ≥10 years diagnosed with RMS had poorer survival than did children aged 1–9. Punyko et al. (2005) reported that the 5‐year survival probabilities were found to be highest for younger age children (ages 1–4 years: 77%). In this study, we found that patients with age >3 years had better OS rate (69.0%) compared with age ≤3 years (42.3%). Among the 26 patients with age ≤3 years, 12 were diagnosed <1 year old. Three children <1 year old were delayed in treatment due to misdiagnosis, which led to the invasion of the lesions to the para‐meningeal. In addition, all of the three patients had residual margin in the first operation and did not receive radiotherapy. Among the 72 patients with age >3 years, 14 were diagnosed after 10 years old (13 cases were in the clinical group III). Combining these factors, children over 3 years old had better prognosis than children under 3 years old. Prognostic factors previously reported also include primary tumor site, tumor size, stage of disease, and histologic tumor subtype (Perez et al., 2011; Simon et al., 2002). In this study, we found that tumor site and pathology type did not predict survival in patients with head and neck RMS. In addition, we found that there were no statistically significant differences in disease‐specific survival were identified when the study group was divided by diagnosis time (≤2010 and >2010 year). However, we found that chemotherapy efficacy was significant predictors of disease‐specific survival. CR was associated with a longer disease‐specific survival (OS rate, 86.6%) compared with PR (OS rate, 6.7%).

Recently, the APBSCT had been widely used in tumor therapy. It was safer and more effective than allograft because of less rejection, easy material collection, and faster recovery of bone marrow hematopoiesis (Kamimura et al., 2011; Niwa et al., 2009; Uehara, Yokota, Onoda, Yamamoto, & Terano, 2008). The key point of high‐dose chemotherapy combined with APBSCT was to kill tumor cells and improve the complete remission rate to achieve long‐term survival (Raney et al., 2002). In this study, three patients received APBSCT treatment and two achieved CR. More importantly, these two patients had a long disease‐free survival even after transplantation. Although one case died after recurrence, the total survival time reached 29 months. Therefore, high‐dose chemotherapy combined with APBSCT for RMS without CR has positive significance. In the future, we should continue to collect cases and clarify their clinical application value.

Although our results suggested that comprehensive treatment could highly increase the clinical remission rate for head and neck RMS patients, the high recurrence rate could not be neglected. Therefore, early diagnosis, comprehensive treatment, and close follow‐up were strongly recommended to improve the clinical remission rate and reduce the recurrence rate and mortality.

In conclusion, improvement in outcome was obtained with comprehensive treatment for head and neck RMS. Both chemotherapy efficacy and age of patients were prognostic factors for children with head and neck RMS, which provide some valuable information for further treatment.

## CONFLICT OF INTEREST

The authors declare that they have no conflict of interest.

## AUTHORS' CONTRIBUTIONS

YZ, WZ, and DH contributed to conception and design; HH and YM data collection; TZ and YM contributed to data analysis and interpretation; YZ, WZ, and YW contributed to providing materials and samples; YZ and WZ contributed to drafting article; DH contributed to approval of article; DH contributed to administrative support. All the authors have read and approved the final manuscript.

## Data Availability

All data generated or analyzed during this study are included in this published article.

## References

[brb31697-bib-0001] Badr, M. , Al‐Tonbary, Y. , Mansour, A. , Hassan, T. , Beshir, M. , Darwish, A. , & El‐Ashry, R. (2012). Epidemiological characteristics and survival studies of rhabdomyosarcoma in East Egypt: A five‐year multicenter study. ISRN Oncology, 2012, 1–8. 10.5402/2012/674523 PMC3362855 22675642

[brb31697-bib-0002] Chen, E. , Ricciotti, R. , Futran, N. , & Oda, D. (2017). Head and neck rhabdomyosarcoma: Clinical and pathologic characterization of seven cases. Head and Neck Pathology, 11(3), 321–326. 10.1007/s12105-016-0771-0 27896667 PMC5550390

[brb31697-bib-0003] Christopher, D. , Fletcher, J. A. , & Krishnan, U. (2013). WHO classification of tumours of soft tissue and bone (4th ed., pp. 110–111). Lyon, France: International Agency for Research on Cancer.

[brb31697-bib-0004] Córdoba, S. R. , & Inarejos, E. C. (2016). Childhood rhabdomyosarcoma. Radiologia, 58(6), 481–490. 27810092 10.1016/j.rx.2016.09.003

[brb31697-bib-0005] Crist, W. M. , Garnsey, L. , Beltangady, M. S. , Gehan, E. , Ruymann, F. , Webber, B. , … Maurer, H. M. (1990). Prognosis in children with rhabdomyosarcoma: A report of the intergroup rhabdomyosarcoma studies I and II. Intergroup Rhabdomyosarcoma Committee. Journal of Clinical Oncology, 8(3), 443–452. 10.1200/jco.1990.8.3.443 2407808

[brb31697-bib-0006] Dasgupta, R. , Fuchs, J. , & Rodeberg, D. (2016). Rhabdomyosarcoma. Seminars in Pediatric Surgery, 25(5), 276–283. 10.1053/j.sempedsurg.2016.09.011 27955730

[brb31697-bib-0007] Huang, D.‐S. , Tang, S.‐Q. , Wang, J.‐W. , Liu, Y. , Liu, L.‐Z. , Yang, G. , & Lv, S.‐G. (2004). Therapeutic effects of the CEM conditioning regimen combined autologous peripheral blood stem cell transplantation on advanced neuroblastoma. Chinese Journal of Practical Pediatrics, 19(9), 9.

[brb31697-bib-0008] Huang, D.‐S. , & Zhang, Y. (2012). Diagnosis and treatment of rhabdomyosarcoma in children. Journal of Clinical Pediatrics, 3, (5), 404–407.

[brb31697-bib-0009] Joshi, D. , Anderson, J. R. , Paidas, C. , Breneman, J. , Parham, D. M. , & Crist, W. (2004). Age is an independent prognostic factor in rhabdomyosarcoma: A report from the Soft Tissue Sarcoma Committee of the Children's Oncology Group. Pediatric Blood & Cancer, 42(1), 64–73. 10.1002/pbc.10441 14752797

[brb31697-bib-0010] Kamimura, T. , Miyamoto, T. , Nagafuji, K. , Numata, A. , Henzan, H. , Takase, K. , … Harada, M. (2011). Role of autotransplantation in the treatment of acute promyelocytic leukemia patients in remission: Fukuoka BMT Group observations and a literature review. Bone Marrow Transplantation, 46(6), 820 10.1038/bmt.2010.207 20818443

[brb31697-bib-0011] Lawrence, W. Jr , Anderson, J. R. , Gehan, E. A. , & Maurer, H. (1997). Pretreatment TNM staging of childhood rhabdomyosarcoma: A report of the Intergroup Rhabdomyosarcoma Study Group. Cancer, 80(6), 1165–1170. 9305719

[brb31697-bib-0012] Malempati, S. , & Hawkins, D. S. (2012). Rhabdomyosarcoma: Review of the Children's Oncology Group (COG) Soft‐Tissue Sarcoma Committee experience and rationale for current COG studies. Pediatric Blood & Cancer, 59(1), 5–10. 10.1002/pbc.24118 22378628 PMC4008325

[brb31697-bib-0013] Maurer, H. M. , Crist, W. , Lawrence, W. , Ragab, A. H. , Raney, R. B. , Webber, B. , … Gehan, E. A. (1988). The intergroup rhabdomyosarcoma study‐I. A final report. Cancer, 61(2), 209–220. 3275486 10.1002/1097-0142(19880115)61:2<209::aid-cncr2820610202>3.0.co;2-l

[brb31697-bib-0014] Niwa, A. , Umeda, K. , Awaya, T. , Yui, Y. , Matsubara, H. , Hiramatsu, H. , … Nakahata, T. (2009). Successful autologous peripheral blood stem cell transplantation with a double‐conditioning regimen for recurrent hepatoblastoma after liver transplantation. Pediatric Transplantation, 13(2), 259–262. 10.1111/j.1399-3046.2008.00948.x 18444949

[brb31697-bib-0015] Okcu, F. , Hicks, J. , & Horowitz, M. (2016). Rhabdomyosarcoma in childhood and adolescence: Epidemiology, pathology, and molecular pathogenesis. Up to date. Retrieved from http://www.uptodate.com/contents/rhabdomyosarcoma‐in‐childhood‐and‐adolescence‐epidemiology‐pathology‐and‐molecularpathogenesis

[brb31697-bib-0016] Perez, E. A. , Kassira, N. , Cheung, M. C. , Koniaris, L. G. , Neville, H. L. , & Sola, J. E. (2011). Rhabdomyosarcoma in children: A SEER population based study. Journal of Surgical Research, 170(2), e243–e251. 10.1016/j.jss.2011.03.001 21529833

[brb31697-bib-0017] Punyko, J. A. , Mertens, A. C. , Baker, K. S. , Ness, K. K. , Robison, L. L. , & Gurney, J. G. (2005). Long‐term survival probabilities for childhood rhabdomyosarcoma: A population‐based evaluation. Cancer, 103(7), 1475–1483. 10.1002/cncr.20929 15712283

[brb31697-bib-0018] Raney, R. B. , Meza, J. , Anderson, J. R. , Fryer, C. J. , Donaldson, S. S. , Breneman, J. C. , … Crist, W. M. (2002). Treatment of children and adolescents with localized parameningeal sarcoma: Experience of the Intergroup Rhabdomyosarcoma Study Group protocols IRS‐II through‐IV, 1978–1997. Medical and Pediatric Oncology, 38(1), 22–32. 10.1002/mpo.1259 11835233

[brb31697-bib-0019] Rodeberg, D. , & Paidas, C. (2006). Childhood rhabdomyosarcoma. Seminars in Pediatric Surgery, 15(1), 57–62. 10.1053/j.sempedsurg.2005.11.009 16458847

[brb31697-bib-0020] Salman, M. , Tamim, H. , Medlej, F. , El‐Ariss, T. , Saad, F. , Boulos, F. , … Saab, R. (2012). Rhabdomyosarcoma treatment and outcome at a multidisciplinary pediatric cancer center in Lebanon. Pediatric Hematology and Oncology, 29(4), 322–334. 10.3109/08880018.2012.676721 22568795

[brb31697-bib-0021] Simon, J. H. , Paulino, A. C. , Smith, R. B. , & Buatti, J. M. (2002). Prognostic factors in head and neck rhabdomyosarcoma. Head and Neck, 24(5), 468–473. 10.1002/hed.10070 12001077

[brb31697-bib-0022] Stiller, C. , Stevens, M. , Magnani, C. , Corazziari, I. , EUROCARE Working Group (2001). Survival of children with soft‐tissue sarcoma in Europe since 1978: Results from the EUROCARE study. European Journal of Cancer, 37(6), 767–774. 10.1016/S0959-8049(01)00007-7 11311652

[brb31697-bib-0023] Tang, S. , Huang, D. , Wang, J. , Wei, X. , Ran, C. , Peng, Y. , … Zhang, J. (2001). Application of autologous peripheral blood stem cell transplantation in children with malignant tumor. Chinese Medical Journal‐Beijing‐English Edition, 114(10), 1098–1101. 11677775

[brb31697-bib-0024] Turner, J. H. , & Richmon, J. D. (2011). Head and neck rhabdomyosarcoma: A critical analysis of population‐based incidence and survival data. Otolaryngology‐Head and Neck Surgery, 145(6), 967–973. 10.1177/0194599811417063 21873599

[brb31697-bib-0025] Uehara, T. , Yokota, A. , Onoda, M. , Yamamoto, K. , & Terano, T. (2008). Successful autologous peripheral blood stem cell transplantation for a patient with primary adrenal lymphoma with hemophagocytic syndrome. Clinical Lymphoma and Myeloma, 8(3), 184–187. 10.3816/CLM.2008.n.024 18650184

[brb31697-bib-0026] Wexler, L. H. , Meyer, W. , & Helman, L. (2011). Rhabdomyosarcoma. Principles and Practice of Pediatric Oncology, 6, 923–953.

[brb31697-bib-0027] Zhang, W.‐L. , Zhang, Y. I. , Huang, D.‐S. , Guo, F. , Han, T. , Hong, L. , … Zhi, T. (2013). Clinical character of pediatric head and neck rhabdomysarcomas: A 7‐year retrospective study. Asian Pacific Journal of Cancer Prevention, 14(7), 4089–4093. 10.7314/apjcp.2013.14.7.4089 23991958

